# Identification of novel microRNAs in *Hevea brasiliensis *and computational prediction of their targets

**DOI:** 10.1186/1471-2229-12-18

**Published:** 2012-02-13

**Authors:** Virginie Gébelin, Xavier Argout, Worrawat Engchuan, Bertrand Pitollat, Cuifang Duan, Pascal Montoro, Julie Leclercq

**Affiliations:** 1 CIRAD, UMR AGAP, F-34398 Montpellier, France; 2 King Mongkut's University of Technology, Thonburi, Thailand; 3 CATAS, RRI, Danzhou, 571737 Hainan, China

**Keywords:** Gene expression, miRNA, *MIR *gene, Next-generation sequencing, Rubber tree, Transcription, Transcriptome, Abiotic stress, miRNA editing

## Abstract

**Background:**

Plants respond to external stimuli through fine regulation of gene expression partially ensured by small RNAs. Of these, microRNAs (miRNAs) play a crucial role. They negatively regulate gene expression by targeting the cleavage or translational inhibition of target messenger RNAs (mRNAs). In *Hevea brasiliensis*, environmental and harvesting stresses are known to affect natural rubber production. This study set out to identify abiotic stress-related miRNAs in *Hevea *using next-generation sequencing and bioinformatic analysis.

**Results:**

Deep sequencing of small RNAs was carried out on plantlets subjected to severe abiotic stress using the Solexa technique. By combining the LeARN pipeline, data from the Plant microRNA database (PMRD) and *Hevea *EST sequences, we identified 48 conserved miRNA families already characterized in other plant species, and 10 putatively novel miRNA families. The results showed the most abundant size for miRNAs to be 24 nucleotides, except for seven families. Several *MIR *genes produced both 20-22 nucleotides and 23-27 nucleotides. The two miRNA class sizes were detected for both conserved and putative novel miRNA families, suggesting their functional duality. The EST databases were scanned with conserved and novel miRNA sequences. MiRNA targets were computationally predicted and analysed. The predicted targets involved in "responses to stimuli" and to "antioxidant" and "transcription activities" are presented.

**Conclusions:**

Deep sequencing of small RNAs combined with transcriptomic data is a powerful tool for identifying conserved and novel miRNAs when the complete genome is not yet available. Our study provided additional information for evolutionary studies and revealed potentially specific regulation of the control of redox status in *Hevea*.

## Background

*Hevea brasiliensis*, the sole commercial source of natural rubber, is a tropical perennial species native to the Amazon basin. South-East Asian countries supplied 92% of the 10 million tons of natural rubber produced in 2010, but ever-growing worldwide demand calls for enhanced tree productivity. This goal is increasingly becoming a crucial challenge for the corresponding research activities in a context of global climate change and redistribution of land for food crops. Natural rubber is a cis-1,4 polyisoprene polymer biosynthesized in rubber particles located in specialized latex cells. Latex cells are periodically emitted from the cambium and then anastomosed to form latificer mantels [1]. Natural rubber is harvested by tapping. The cytoplasm of laticifers, containing 30-50% of rubber particles, is then expelled by high turgor pressure maintained in the soft bark tissues. An ethylene generator, 2-chloroethylphosphonic acid (ethephon), is applied to the tapping panel to stimulate latex production. Ethephon increases the duration of latex flow after tapping and its regeneration between two tappings [2]. Tapping Panel Dryness (TPD) is a physiological disease that causes 10-40% annual rubber production losses over the 30 years of a rubber cultivation cycle. This physiological disorder, triggered by oxidative stress, is partially induced by excessive environmental and harvesting stresses [3]. The generation of reactive oxygen species (ROS) in latex cells leads to in situ coagulation of rubber particles [4,5]. Research on ROS in plants initially focused on their cytotoxicity only, but nowadays ROS are also considered as signalling molecules [6,7]. A ROS signalling network emerges and is conserved in all aerobic organisms [8]. The ROS-scavenging system acts both to maintain redox homeostasis and to protect cell components from oxidative damage. Biotic and abiotic stress, such as drought, salinity, strong light, temperature, heavy metals, UV radiation, atmospheric contamination, mechanical wounding, nutrient starvation and pathogen attacks are major sources of ROS in plants [9].

MicroRNAs (miRNAs) particularly play a key function in responses to abiotic stress (for a review see [10-13]). Indeed, small RNAs are involved in fine-tuning gene expression in response to physiological, external and developmental stimuli [14,15]. Of these, miRNAs have been shown to be a large group of small endogenous RNAs that exist in animals, plants and viruses. Since their discovery in *Caenorrhabditis elegans *[16] and in *Arabidopsis thaliana *in 2002 [17], miRNAs have been described as negatively regulating gene expression by targeting specific messenger RNAs (mRNAs) for cleavage [18,19] or translational inhibition [20]. The biogenesis of miRNAs has been amply described in plants and animals [21-24]. Briefly, a *MIR *gene is first transcribed to primary miRNAs (pri-miRNA) by RNA polymerase II, the 70-500 bp long pri-miRNA forms an imperfectly paired hairpin which is processed by RNAse III-like DICER-like I (DCL1) to generate miR precursors (pre-miRNAs) [25,26]. Further cleavage of the pre-miRNA by DCLI releases a miRNA/miRNA* duplex. The duplex is then translocated into the cytoplasm by HASTY (plant ortholog to exportin 5 [27]), the canonical mature miRNA of 20-22 nucleotides is selectively incorporated into the RNA-induced silencing complex (RISC) associated with Argonaute 1 (AGO1). In the RISC complex, miRNAs bind to mRNA and inhibit gene expression through perfect or near-perfect complementarity between the miRNA and the mRNA [18]. In plants, the recent discovery of *MIR *genes generating both canonical miRNA (20-22 nucleotides) and long miRNA (lmiRNA, 23-27 nucleotides) suggests their bifunctionality [28]. The lmiRNAs derived from *MIR *genes are selectively processed by DCL3, can associate with AGO4 and guide DNA methylation at some of their target loci in *trans *in rice and moss [28,29]. The recent and major discovery of the role played by post-transcriptional modifications of miRNA precursors highlighted the complexity of miRNA biogenesis and the way miRNAs act [30,31]. Indeed, adding an oligo-uridine tail to the 3' end of the miRNA precursor is a mechanism that controls miRNA production in mice [31]. In plants, it has been reported that either a combination of 5' deletion and 3' uridylation of miRNA, or RNA editing events, alter the targeting and preference of Argonaute association [30]. Several miRNAs have been described as being up-regulated or down-regulated by high salinity, drought and low temperatures [32,33]. Furthermore, the miRNA-targeted gene *copper zinc superoxide dismutase *(*CuZnSOD*), which plays a major role in maintaining redox homeostasis, is cleaved by miR398 under stress-free conditions in *Arabidopsis *[34,35].

Currently, 15,172 mature miRNAs have been discovered and deposited in the public database, miRBase (Release 16, 2010, http://www.mirbase.org) [36,37], and 9,277 mature plant miRNAs in the Plant MicroRNA Database, PMRD http://bioinformatics.cau.edu.cn/PMRD[38]. Most miRNAs have been identified in model species such as *Arabidopsis, Oryza *[39,40], *Populus *[12,41], *Physcomitrella *[42], *Vitis *[43], whose genomes have been sequenced. Some miRNAs have also been identified in *Glycine max *[44], *Arachis hypogaea *[45], *Solanum lycopersicum *[46], *Brassica napus *[47], *Phaseolus vulgaris *[48] and in the euphorbiaceous species *Ricinus communis *[49]. Twenty-three miRNA families predicted in *Ricinus communis *were experimentally validated in four euphorbiaceous plants (*Ricinus communis, Jatropha curcas, Manihot esculenta *and *Hevea brasiliensis*) [49]. However, the strategy used did not allow exhaustive identification of miRNA families in *Hevea*.

The availability of large collections of *Hevea *Expressed Sequence Tags (ESTs; http://bassigny/cgi-bin/esttik_dev/quick_search.cgi) and next-generation sequencing techniques offers prospects for understanding the post-transcriptional regulation of key functions involved in responses to stress and in latex production in *Hevea brasiliensis*. This study involved the deep sequencing of small RNAs from *Hevea *plants grown under various conditions. We report here on the identification of 48 conserved miRNA families, sharing very high homology with that already known in other species. MiRNA precursor sequences were identified for nine families. In addition, ten putative novel miRNA families were also identified with their precursors. A scan of the *Hevea *EST databases with miRNA sequences revealed their putative targets. Of them, the predicted targets involved in responses to stimuli, the ROS-scavenging systems and transcription regulation are presented, and new insights into the control of redox status in *Hevea *are proposed.

## Results

### Classification of small RNAs

A total of 4,223,792 raw reads was generated by Solexa sequencing from the small RNA library prepared from pooled juvenile and mature plant materials subjected to various types of abiotic stress (Table 1 and Table 2). After removing adapters, cleaned reads amounted to 2,378,135 sequences corresponding to 670,645 unique sequences. The sequence lengths ranged from 17 to 32 nucleotides. Although some small RNAs were present about a thousand times in our dataset, most were sequenced only a few times. Small RNAs consisting of only one or two reads accounted for 32% and 53% of clean sequences, respectively.

**Table 1:** Statistics of small RNA sequences from the *Hevea brasiliensis *small RNA library

Stress small RNA library	Sequence number	Analysis
read sequences	4,223,792	
	
clean sequences	2,378,135	Solexa sequencing
	
unique clean sequences of small RNA	670,645	

no hit	629,795	
	
hit, unknown	26,721	
	
coding mRNA	6,618	
	
transposons/transposable element	6,800	
	
rRNA	399	Blast against *Arabidopsis*
	
pseudo mRNA	138	genome
	
ncRNA	73	
	
miRNA	57	
	
tRNA	39	
	
sn/snoRNA	5	

no hit	652,215	
	
hit	18,430	Blast against PMRD
	
conserved miRNA family	48	

putative novel miRNA family	10	LeARN pipeline

**Table 2:** List of the treatments applied to *Hevea *plants before small RNA isolation

Treatments	Plant material	Tissue	Condition
Ethylene	in vitro plantlet (2 years old)	leaf, bark, root	5 ppm for 4 hours
	
	budded plant	leaf, bark	

Methyl-jasmonate	in vitro plantlet (2 years old)	leaf, bark, root	0.3 μM for 4 hours
	
	budded plant	leaf, bark	

Wounding	in vitro plantlet (2 years old)	leaf, bark, root	2 hours
	
	budded plant	leaf, bark	

Drought	in vitro plantlet (2 years old)	leaf, bark, root	10 days

	budded plant	leaf, bark	4 weeks

Flooding	in vitro plantlet (2 years old)	leaf, bark, root	4 days
	
	budded plant	leaf, bark	

Cold	in vitro plantlet (2 years old)	leaf, bark, root	4°C for 8 hours
	
	budded plant	leaf, bark	

Light	in vitro plantlet (2 years old)	leaf, bark, root	PAR: 1000-1500 μmol/m^2^/s for
	
	budded plant	leaf, bark	4 hours

NaCl	in vitro plantlet (2 years old)	leaf, bark, root	300 mM for 4 days
	
	budded plant	leaf, bark	

	in vitro plantlet (2 years old)	leaf, bark, root	
	
Control	budded plant	leaf, bark	
	
	in vitro plantlet (1 month old)	leaf, bark, root	
	
	callus	callus	

Annotation of the *Hevea *small RNAs was attempted by BLASTN on the *Arabidopsis *genome. Most of the sequences did not map onto the *Arabidopsis *genome sequence. Of the mapped sequences, 26,721 were not annotated. Annotated sequences predicted 6,618 products of mRNA degradation, 6,800 transposable elements, 399 rRNAs, 138 products of pseudogene degradation, 73 ncRNAs, 39 tRNAs, 5 sn/snoRNAs and 57 miRNAs corresponding to 14 families.

**Figure 1:**
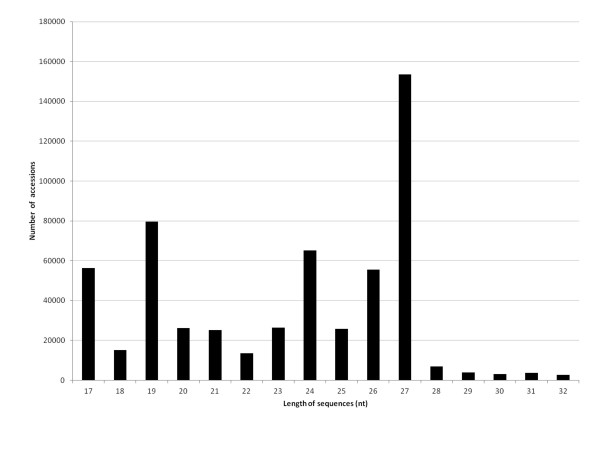
**Length distribution of unique accessions in the small RNA dataset in *Hevea brasiliensis***.

Further mapping was carried out with all the miRNA sequences available in the PMRD database. In all, 18,430 sequences matched (0 or 1 mismatch) to conserved miRNAs in other species. The accessions were classified into 48 miRNA families (Table 1). Since most sequences did not map against PMRD, the *Hevea *EST database was scanned using the LeARN pipeline. This strategy led to the identification of 10 additional miRNA families, based on the stem-loop structure, which were not described in other species (Table 1). The distribution of the 670,645 unique accessions showed that the most abundant accessions had in descending order 27, 19, 24, 26 and 17 nucleotides (Figure 1). Accessions with 17 and 19 nucleotides came from products of mRNA degradation and transposable elements according to the mapping results against the *Arabidopsis *genome sequence (data not shown).

### Characteristics of conserved and putative novel miRNA families in *Hevea brasiliensis*

**Figure 2:**
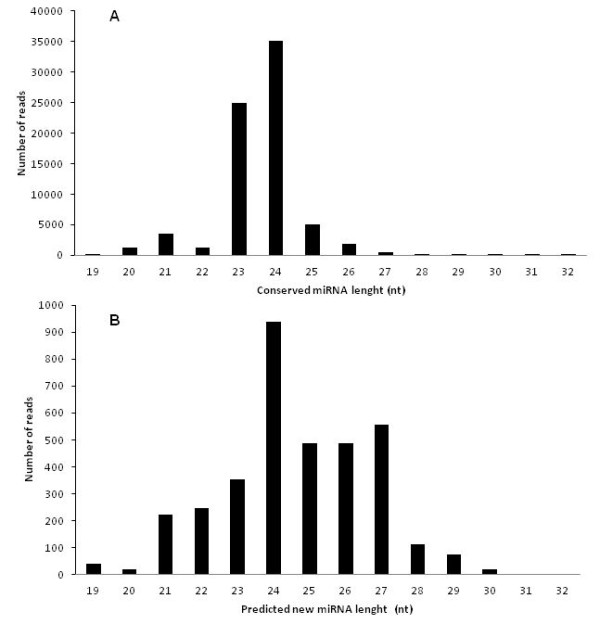
**Length distribution of reads matching miRNA families in *Hevea brasiliensis***. (A) conserved miRNA (B) putatively novel families.

Reads of conserved and putative novel miRNAs were distributed according to their nucleotide lengths (Figure 2). The 48 conserved miRNA families mostly had 24 and 23 nucleotides (Figure 2A). For the 10 putative novel miRNA families, the most represented lengths were 24, 27 and 26 nucleotides (Figure 2B).

**Figure 3:**
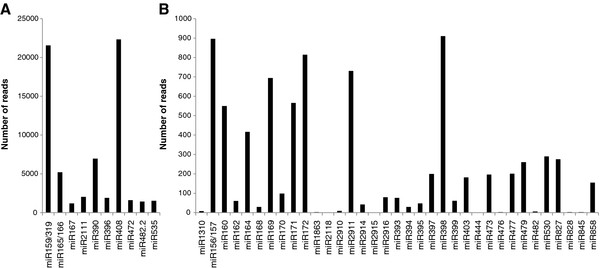
**Abundance of the 48 conserved miRNA families in *Hevea brasiliensis *with 0 and 1 mismatch on a minimum length of 19 nucleotides**. **A**) The sequencing frequency of a conserved miRNA family in the small RNA library above 1000 reads. **B**) The sequencing frequency of a conserved miRNA family in the small RNA library below 1000 reads.

Of the 48 conserved miRNA families, 12 were predominant with more than 1,000 reads, including 3 families that displayed more than 20,000 reads. The latter corresponded to HbmiR159/319 and HbmiR408 (Figure 3A). By contrast, 36 families were less represented in the dataset with 18 families having fewer than 100 reads: HbmiR1310, HbmiR162, HbmiR168, HbmiR1863, HbmiR2118, HbmiR2910, HbmiR2914, HbmiR2915, HbmiR2916, HbmiR393, HbmiR394, HbmiR395, HbmiR399, HbmiR444, HbmiR476, HbmiR482, HbmiR828 and HbmiR845 (Figure 3B).

**Figure 4:**
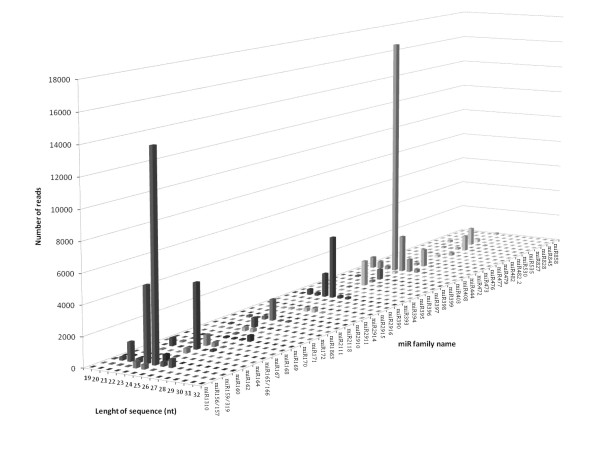
**Number of reads according to the length distribution of accessions of conserved miRNA families in *Hevea brasiliensis***.

**Figure 5:**
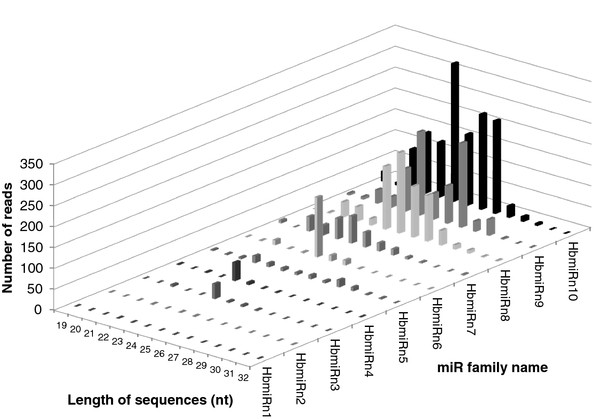
**Number of reads according to the length distribution of accessions of putative novel miRNA families in *Hevea brasiliensis***.

A detailed analysis of length distribution within the *Hevea *miRNA families revealed some discrepancies (Figure 4 and Figure 5). Firstly, the 20-22 nucleotide and 23-27 nucleotide size classes were detected for both conserved and novel miRNA families. Secondly, the most abundant size for miRNAs was 21-24 nucleotides, except for HbmiR169 (27 nucleotides), HbmiR2911 (26 nucleotides), HbmiR482 (25 nucleotides), HbmiR472 (25 nucleotides), HbmiRn8 (25 nucleotides) and HbmiRn9 (27 nucleotides). Thirdly, the putative novel miRNAs occurred at low levels with fewer than 500 reads, and at extremely low levels for HbmiRn1 and HbmiRn2, with only three sequences (Figure 5).

### Detection of single-nucleotide mutations in miRNA sequences

For all the conserved miRNA families, single-nucleotide modifications in the sequences were analysed in detail based on the data presented in [30]. In *Arabidopsis*, seventeen families are concerned by RNA editing events and nine families by 5' deletion and 3' uridylation modifications (called below -1 + UU modification). In *Hevea*, of the seventeen families, four families displayed single-nucleotide mutations at the sites of post-transcriptional RNA modifications described in *Arabidopsis*, namely AtmiR156, AtmiR159a, AtmiR164a, and AtmiR172a (Table 3). By contrast, no -1 + UU modification was observed in *Hevea *(Table 3).

**Table 3:** Frequency of single-nucleotide mutation and of a combination of 5' deletion and 3' uridylation (-1 + UU) for four miRNA families from *Hevea*

Family name in *Arabidopsis*	Single-nucleotide modification		-**1 + UU modification**	
	***Arabidopsis***	***Hevea***	***Arabidopsis***	***Hevea***
	
AtmiR156	G-A: 14.091%	A-U: 49.382% A-C: 3.571%	yes	no

	G-A: 10.407%	G-A: 2.127%		
		
AtmiR159a	G-A: 8.597%	G-A: 4.166%	no	no
		
	G-A: 5.882%	G-C: 2.127% G-U: 1.398%		

AtmiR164a	G-A: 3.967%	G-A: 7.692%	no	no

AtmiR172a	G-U: 0.599%	G-U: 6.153% A-U: 7.692% C-U: 7.692%	no	no

Sites of post-transcriptional modification identified in *Arabidopsis *[50] were analysed in the *Hevea *small RNA dataset. Only sequences present at > 0.5% are listed

### Identification of precursor transcripts for conserved and putative novel miRNA families from the *hevea brasiliensis *transcriptome

**Figure 6:**
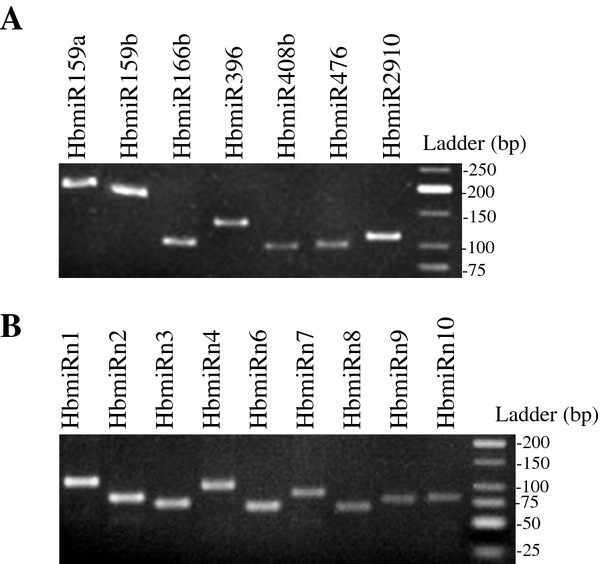
**Gel electrophoresis of stem-loop RT-PCR**. A) Stem-loop RT-PCR from seven precursors of conserved miRNA family. **B**) Stem-loop RT-PCR from nine precursors of putatively new miRNAs.

Conserved and putative novel miRNA precursors were sought in the *Hevea *clone PB260 transcript sequence databases obtained from various organs using the LeARN pipeline [51]. Eight conserved miRNA families that mapped against RNA sequences displayed a stem-loop structure (Table 4 and Additional Table 1). A single miRNA precursor transcript was found for the HbmiR156, HbmiR159, HbmiR396, HbmiR476 and HbmiR2910 families. Two miRNA precursor transcripts were identified for the HbmiR166, HbmiR319 and HbmiR408 families. In addition, the LeARN pipeline predicted 10 putative novel miRNAs with their unique precursors (Table 5 and Additional Table 2). Mapping the sequences of the small RNA dataset on the precursor sequences led to the identification of miRNA*for all eight conserved families (HbmiR156, HbmiR159, HbmiR166, HbmiR319, HbmiR396, HbmiR408, HbmiR476 and HbmiR2910) and for the five putatively new families HbmiRn3, HbmiRn4, HbmiRn8, HbmiRn9 and HbmiRn10. Stem-loop reverse transcription polymerase chain reactions (RT-PCR) were successfully performed on seven precursors for the conserved families and on nine for the putatively new families (Figure 6).

**Table 4:** List of precursors for conserved microRNAs identified in the *Hevea *clone PB260 transcriptome sequences

New miR name	Accession Name	Mature sequence	Mature length	miRNA* (Accession No.)	Precursor length	EST
HbmiRn1	acc_584359	CAGGAACUGGUAUCAACCCAGC	22	nd	130	CL1Contig1853_r1

HbmiRn2	acc_495109	UAUUGUAGAAAUUUUCAGGAUC	22	nd	116	CL1Contig490_r1

HbmiRn3	acc_185377	UAAUGGGCUCUGCAUAGAUGG	21	Yes (3)	108	CL27Contig1_r1

HbmiRn4	acc_108425	UUGCAUAUCUCAGGAGCUUCA	21	Yes (4)	214	CL19922Contig1_r1

HbmiRn5	acc_644211	AAACGGCUACCACAUCCAA	19	nd	87	FYGXE6I01B755W_r1

HbmiRn6	acc_501419	UAGGAUGUAGAAGAGCAUAA	20	nd	90	F5VNCTM02H9XL3_r1

HbmiRn7	acc_35715	UAGUUUGUUUGAUGGUAUC	19	nd	113	FYGXE6I01EKFYA_r1

HbmiRn8	acc_246127	GAUUGACAGACUGAGAGCUC	20	Yes (16)	93	FYGXE6I01B7GPL_r1

HbmiRn9	acc_103082	UUUAUGAAAGACGAACAACUG	21	Yes (6)	87	CL1Contig2798_r1

The presence of the miRNA* sequence in the small RNA dataset is indicated. Pictures of the stem-loop structures are presented in Additional Table 1.

**Table 5:** List of putatively new miRNA precursors identified in the *Hevea *clone PB260 transcriptome sequences

MiRNA name	Accession Name	Mature sequence	Mature length	miRNA* (Accession No.)	Precursor length	EST
Hbmir156	acc_462135	UUGACAGAAGAUAGAGAGC	19	Yes (10)	119	FYGXE6I01DJH4X_r1

Hbmir159	acc_17403	UUUGGAUUGAAGGGAGCUCUA	21	Yes (5)	221	GETTCZF01AWUSL_r1

Hbmir166	acc_28021	UCGGACCAGGCUUCAUUCC	19	Yes (14)	127	CL2671Contig1_r1
	
	acc_150486	UCGGACCAGGCUUCAUUCCCCC	22	Yes (14)	120	CL1Contig15359_r1

Hbmir319	acc_19786	UUGGACUGAAGGGAGCUCCCU	21	Yes (7)	221	FRZQES201D34W3_r1

Hbmir396	acc_112787	UUCCACAGCUUUCUUGAACUG	21	Yes (15)	154	CL1Contig13811_r1

Hbmir408	acc_393516	AAGACUGGGAACAGGCAGAGCA	22	Yes (294)	128	FYGXE6I01CVDV9_r1
	
	acc_39581	ACUGGGAACAGGCAGAGCAUGA	22	Yes (294)	120	CL3908contig1_r1

Hbmir476	acc_508145	UAAUCCUUCUUUGCAAAGUC	20	Yes (1)	126	CL1Contig11471_r1

Hbmir2910	acc_251816	GAGCGAUUUGUCUGGUUAAUC	21	Yes (5)	126	FYGXE6I01EDXGX_r1

The presence of the miRNA* sequence in the small RNA dataset is indicated (nd: not detected). Pictures of the stem-loop structures are presented in Additional Table 2.

### Comparison of the gene ontology classification of predicted targets for conserved and putative novel miRNA families

**Figure 7:**
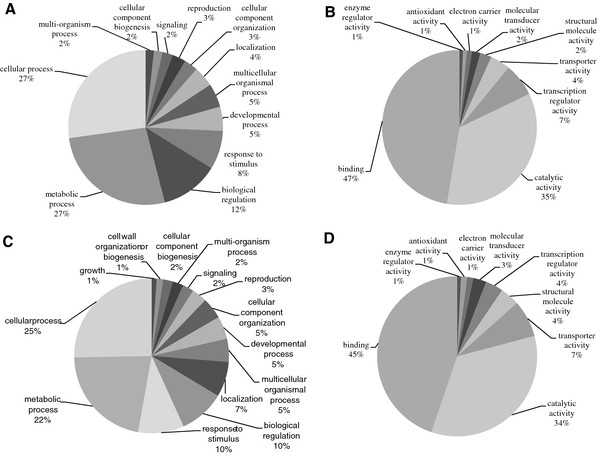
**Pie chart representation of Gene Ontology terms at level 2 for putative targets of conserved (A and B) and novel miRNAs (C and D)**. A and C) Gene Ontology terms for biological processes, B and D) Gene Ontology terms for molecular functions.

Computational prediction of miRNA targets led to the identification of 1,083 sequences for the 48 conserved families and 705 for the 10 putative novel miRNA families. Their involvement in biological processes and their molecular functions were attributed using Gene Ontology (Figure 7A, B, C and 7D). Most of the GO terms were represented in the same proportions for predicted targets from conserved and putative novel miRNA families. Target genes of the putative novel miRNA families all had GO terms of conserved miRNA families for biological processes and molecular functions. In biological process terms, the putative novel miRNA families had two additional terms (growth, cell wall organization or biogenesis). The number of miRNA target genes was smaller for putative novel than for conserved miRNAs for some GO terms: cellular process (25% as opposed to 27%), metabolic process (22% as opposed to 27%) and biological regulation (10% as opposed to 12%) (Figures 7A and 7C). This number was larger for putative novel miRNA targets compared to conserved targets for cellular component organization (5% as opposed to 3%), localization (7% as opposed to 4%) and response to stimulus (10% as opposed to 8%) (Figures 7A, B, C and 7D). As regards the GO terms for molecular function, although the distribution for antioxidant activity was the same for the 2 classes of miRNAs (1%), a decrease in the proportion of transcription regulator activity (4% as opposed to 7%) and an increase in structural molecule activity (4% as opposed to 2%) and transporter activity (7% as opposed to 4%) were observed for the putative novel miRNA families (Figures 7B and 7D).

### Comparison of predicted target genes for conserved and putative novel miRNA families involved in "response to stimulus", and in "antioxidant" and "transcription regulation" activities

As the management of oxidative stress is crucial for rubber productivity, we set out to more effectively understand the mechanisms of redox homeostasis. We therefore gave priority to predicted miRNA-targeted genes involved in the "response to stimulus" (biological process) and in "antioxidant" and "transcription regulator" activities (molecular process) according to the GO annotations (Tables 6 and 7).

**Table 6:** List of predicted targets for conserved miRNAs involved in response to stimulus, and in antioxidant and transcription regulator activities according to GO terms

MiRNA family	GO term	Predicted target function	mfe kcal/mol	Target name
	Transcription regulation activity	Squamosa promoter-binding protein	-45.50	CL2120Contig2
	
HbmiR156/157		APETALA2-like protein	-24.41	hevea_454_rep_c24306
	
	Antioxidant activity	flavonoid 3',5'-hydroxylase	-26.53	CL2495Contig2
	
		AKR (aldo keto reductase)	-32.90	hevea_454_rep_c15059

	Transcription regulation activity	APETALA2-like protein	-27.80	hevea_454_rep_c17780
	
		sarcosine oxidase	-29.70	CL11607Contig1
	
		serine/threonine protein kinase	-21.39	CL11Contig15
	
		cinnamoyl-CoA reductase	-32.88	CL1Contig7212
	
		CuZnSOD peroxysomal	-32.14	CL1Contig3818
	
HbmiR159	Antioxidant activity	ABC transporter C family	-28.02	CL1Contig3612
	
		cytochrome b6f complex	-36.18	CL1Contig475
	
		TATA box binding protein	-29.38	CL1Contig760
	
		oxidoreductase family protein	-32.88	CL781Contig2
	
		ATP Synthase	-27.31	CL1613Contig1
	
		HMG-CoA_reductase (HMGR)	-36.39	CL8048Contig1
	
		6-phosphogluconate dehydrogenase family protein	-33.44	CL1Contig1588

HbmiR319	Antioxidant activity	ferritin putative	-35.86	CL1Contig17258

HbmiR160	Transcription regulation activity	ARF	-52.20	CL6582Contig1

	Antioxidant activity	FAD/NAD(P)-binding oxidoreductase-like protein	-23.10	hevea_454_rep_c15774
	
HbmiR162		ABC transporter C family	-26.47	hevea_454_rep_c28843
	
	Response to stimulus	zinc finger family protein	-31.40	hevea_454_rep_c20758

HbmiR164	Transcription regulation activity	NAC-domain protein	-44.12	hevea_454_rep_c10543

		amine oxidase	-32.67	CL21795Contig1
	
	Transcription regulation activity	NAC-domain protein	-32.43	CL7990Contig1
	
		PHAVOLUTA-like HD-ZIPIII protein	-46.68	hevea_454_rep_c12680
	
HbmiR165/166	Antioxidant activity	LRR protein	-30.08	hevea_454_c28164
	
	Antioxidant activity/Response to stimulus	malate deshydrogenase	-38.95	hevea_454_rep_c94776
	
	Response to stimulus	abscisic acid insensitive protein	-36.33	hevea_454_rep_c69184

Hbmir167	Response to stimulus	cap binding protein	-33.60	hevea_454_c94262

	Transcription regulation activity	CCAAT-binding transcription factor	-32.79	CL435Contig5
	
HbmiR169		Laccase	-26.51	CL1Contig13876
	
	Antioxidant activity	aldehyde dehydrogenase	-26.56	CL1Contig16398
	
		annexin-like protein	-32.71	CL1Contig2060

HbmiR170	Transcription regulation activity	GRAS domain-containing protein	-37.90	hevea_454_rep_c11063

	Transcription regulation activity	GRAS domain-containing protein	-39.50	CL3142Contig4
	
HbmiR171		ribonucleoside-diphosphate reductase	-26.99	CL1031Contig2
	
	Antioxidant activity	oxophytodienoate reductase (OPR)	-27.23	CL3790Contig2
	
		glyceraldehyde-3-phosphate dehydrogenase	-24.88	CL150Contig1

	Transcription regulation activity	APETALA2-like protein	-24.25	hevea_454_rep_c45080
	
		HD-ZIP protein	-33.63	hevea_454_rep_c13497
	
HbmiR172	Antioxidant activity	NAD(P)H-quinone oxidoreductase	-24.54	hevea_454_rep_c13256
	
		CLAVATA1 putative	-31.73	hevea_454_rep_c2026
	
		Squalene monooxygenase	-29.04	hevea_454_rep_c32943
	
	Response to stimulus	syntaxin	-31.00	hevea_454_c68550
	
		aspartate aminotransferase	-33.44	hevea_454_rep_c4007

HbmiR2111	Antioxidant activity	laccase	-26.57	hevea_454_rep_c5912

HbmiR2910	Antioxidant activity	2OG-Fe(II) -dependent-oxygenase-like protein	-30.83	hevea_454_rep_c1596
	
		ketol-acid reductoisomerase	-31.31	hevea_454_rep_c5152

HbmiR2914	Antioxidant activity	FAD Binding domain-containing protein	-58.64	hevea_454_rep_c15793
	
		Rboh	-31.88	hevea_454_rep_c23550

	Transcription regulation activity	Heat shock protein	-30.89	CL11591Contig1
	
Hbmir390		WRKY transcription factor	-33.09	CL338Contig4
	
	Antioxidant activity	Phosphoenolpyruvate carboxylase	-19.81	CL362Contig2
	
		hydroxy-acid oxidase	-23.58	CL8468Contig1

HbmiR393	Transcription regulation activity	APETALA2-like protein	-22.79	hevea_454_c60993

	Transcription regulation activity	APETALA2-like protein	-24.47	hevea_454_c37716
	
Hbmir395	Antioxidant activity	2OG-Fe(II) -dependent-oxygenase-like protein	-30.00	hevea_454_rep_c21129

	Transcription regulation activity	APETALA2-like protein	-22.40	hevea_454_rep_c13430
	
		Transparent TESTA protein	-34.16	hevea_454_rep_c14652
	
		GRAS domain-containing protein	-27.01	hevea_454_rep_c8477
	
HbmiR396	Antioxidant activity	zinc finger family protein	-31.84	hevea_454_c64598
	
		Lignin-forming anionic peroxidase	-26.08	hevea_454_rep_c20896
	
	Antioxidant activity/Response to stimulus	Glutamyl-tRNA reductase	-35.76	hevea_454_rep_c14169
	
		fatty acyl-coenzyme A reductases (FAR)	-25.41	hevea_454_rep_c16435
	
		cysteine protease	-33.18	CL1Contig374

HbmiR397	Antioxidant activity	laccase	-40.97	hevea_454_c73766

	Antioxidant activity	4-coumarate-coa ligase	-37.60	hevea_454_rep_c6270
	
HbmiR398		CuZnSOD chloroplastique	-37.30	CL4308Contig2
	
	Response to stimulus	HVA22-like protein	-36.03	hevea_454_rep_c27557

	Transcription regulation activity	APETALA2-like protein	-28.43	hevea_454_rep_c64305
	
		serine/threonine protein kinase	-32.93	CL23251Contig1
	
		Heat shock protein	-26.98	CL530Contig7
	
		cytochrome P450	-37.63	CL1Contig8196
	
HbmiR408		enolase	-42.49	CL5843Contig1
	
	Antioxidant activity	ribonucleoside-diphosphate reductase	-37.34	CL1031Contig6
	
		thioredoxin reductase	-32.80	CL1401Contig3
	
		gibberellin 20-oxidase	-37.63	CL1Contig12612
	
		4-hydroxy-3-methylbut-2-enyl diphosphate reductase	-30.00	CL1Contig6885

HbmiR444	Transcription regulation activity	WRKY	-36.82	hevea_454_rep_c7550

		Heat shock protein	-29.45	CL2547Contig3
	
HbmiR476	Antioxidant activity	Rboh	-27.76	CL1Contig1292
	
		cationic peroxidase 2 precursor	-31.63	CL257Contig2

HbmiR535	Antioxidant activity	LRR protein	-29.18	hevea_454_c18080

HbmiR827	Response to stimulus	fructose-bisphosphate aldolase	-26.90	hevea_454_rep_c59114

HbmiR828	Transcription regulation activity	MYB class transcription factor	-26.76	hevea_454_rep_c48615

**Table 7:** List of predicted targets for putative novel miRNAs involved in response to stimulus, and in antioxidant and transcription regulator activities according to GO terms

miRNA family	Accession number	GO term	Predicted target function	mfe kcal/mol	Target name
HbmiRn1	Acc_584359	Response to stimulus	Aquaporin like protein	-47.70	CL1Contig2288
	
		Transcription regulation activity	Helicase like protein	-31.10	CL18570Contig1

HbmiRn2	Acc_495109	Response to stimulus	glycogen synthase kinase	-21.10	CL1Contig2247
	
		Transcription regulation activity	TAZ zinc finger	-22.40	CL5132Contig2

HbmiRn3	Acc_185377	Transcription regulation activity	MYC	-27.14	CL1Contig2024

HbmiRn4	Acc_108425	Transcription regulation activity	bZIP domain trnscription factor	-29.10	CL11387Contig1

			Peroxidase	-28.30	CL1Contig15444
	
		Response to stimulus	gamma-glutamylcysteine synthetase	-27.80	CL786Contig2
	
HbmiRn5	Acc_644211		CTR1	-29.18	CL3295Contig2
	
		Transcription regulation activity	Auxin-responsive protein	-29.20	CL16079Contig1
	
			GRAS family protein	-33.17	CL2606Contig1

HbmiRn6	Acc_501419	Response to stimulus	ubiquitin fusion protein	-37.74	CL1Contig4048

		Response to stimulus	heat shock protein	-27.50	CL4213Contig3
	
			heat shock protein	-23.79	CL1Contig6482
	
			glycerol kinase	-26.20	CL6130Contig1
	
HbmiRn7	Acc_35715		glycine-rich RNA-binding protein	-22.90	CL2515Contig2
	
			ubiquitin-protein ligase	-21.10	CL7863Contig1
	
		Transcription regulation activity	NAC domain protein	-27.06	CL678Contig1
	
			LRR receptor-like protein kinase	-26.16	CL6991Contig1

HbmiRn8	Acc_246127	Response to stimulus	SAUR family protein	-27.40	CL3428Contig1

HbmiRn9	Acc_103082	Response to stimulus	Peroxidase	-35.30	CL1Contig7525

HbmiRn10	Acc_482742	Response to stimulus	calcium-dependent protein kinase	-23.30	CL1Contig9241
	
			longevity assurance factor	-22.15	CL8027Contig1

Twelve members of the AP2/ERF domain-containing transcription factor family were putatively targeted by HbmiR156, HbmiR159, HbmiR172, HbmiR393, HbmiR395, HbmiR396, and HbmiR408. Two HD-ZIP III domain-containing protein members (HbmiR165/166, HbmiR172), a transcription factor with a bZIP domain (HbmiRn4), three NAC domain-containing proteins (HbmiR164, HbmiR165 and HbmiRn7), three Squamosa promoter binding proteins (HbmiR156/157), four WRKY transcription factors (HbmiR156/157, HbmiR390, HbmiR444), four Auxin Response Factor (ARF); HbmiR160 and HbmiRn5) and five GRAS family transcription factors (HbmiR170, HbmiR171, HbmiR396 and HbmiRn5) were also potentially targeted.

Sequences encoding protein involved in protein degradation, such as cysteine protease (HbmiR396) and ubiquitin protein ligase (HbmiRn6 and HbmiRn7), were identified for genes classified in the "response to stimulus" category.

Three sequences related to ABA signalling were also identified: an ABA-insensitive protein (HbmiR166, and HbmiR2910), and an HVA22-like protein (HbmiR398). In addition, one sequence encoding CTR1, a negative regulator of the ethylene signalling pathway, was targeted by HbmiRn5. MiRNA biogenesis was also highlighted by the presence of a cap binding protein predicted to be targeted by HbmiR167. Interestingly, two enzymes involved in natural rubber biosynthesis, HMG-CoA reductase and 4-hydroxyl-3-methylbut-2-enyl diphosphate reductase, were predicted to be targeted by HbmiR159 and HbmiR444, respectively. For the "antioxidant activity" category, several ROS-scavenging enzymes were identified. The chloroplastic and peroxisomal isoforms of CuZnSOD were putatively targeted by HbmiR398 and HbmiR159, respectively. Another enzyme involved in glutathione biosynthesis, gamma glutamyl cysteine ligase (GCL), was predicted to be targeted by HbmiRn5. Rboh (or NADPH oxidase) is an enzyme involved in ROS production, which is likely to be targeted by two miRNAs (HbmiR2914 and HbmiR476). In addition, targets that are subjected to or are involved in the redox status of proteins, such as genes encoding proteins involved in lignin synthesis, cynnamoyl CoA reductase, lignin forming anionic peroxidase precursor and 4-coumarate:CoA ligase, were also found to be targeted by HbmiR159, HbmiR408 and HbmiR398, respectively.

The cleavage site for three targets has been experimentally validated for a chloroplastic CuZnSOD, a Squamosa promoter binding protein and an ARF (Table 8). For these targets, the cleavage site is located at the canonical 10th nucleotide.

**Table 8:** List of target genes experimentally cleaved by miRNA

miRNA family	Target name				*Hevea *sequence name	miRNA accession	mfe kcal/mol	Alignment			
Hbmir156	Squamosa promoter binding protein (HbSquamosa)				CL2120Contig2	acc_480780	-45.5	miRNA Target	23 766	CUACACGAGAGAGAGAAGACAGU : ::::::::::::::::::::: GUUGUGCUCUCUCUCUUCUGUCA ▲ 8/14	1 788

Hbmir160	Auxin Response Factor (HbARF)				CL6582Contig1	acc_370	-52.2	miRNA Target	21 524	ACCGUAUGUCCCUCGGUCCGU ::::::.:::::::::::::: UGGCAUGCAGGGAGCCAGGCA ▲ 27/30	1 544

HbmiR398	Chloroplastic copper zinc superoxide dismutase (HbCuZnSODchl)				CL4308Contig2	acc_420	-37.3	miRNA Target	23 456	UAGU-C-CCCGCUGGACUCU-UGUGU .::: : :::.::::::.:: :::.: GUCAUGCGGGUGACCUGGGAAACAUA ▲ 6/14	1 480

The number of sequences presenting the same cleavage site in indicated below the cleavage site located at the 10th nucleotide (arrow)

## Discussion

### Size distribution of small RNAs in *Hevea *may differ from others species

Next-generation high-throughput sequencing techniques are powerful tools for miRNA identification. The sequencing and analysis of the *Hevea *small RNA library generated more than 2 million sequences. In their vast majority (86%), the sequenced small RNAs were only represented by one or two reads. Similarly, 65% of unique miRNA sequences were found in *Arabidopsis *by 454 sequencing techniques [52]. Interestingly, we found that the size distribution of *Hevea *small RNAs differed from other species. The most abundant small RNAs had 17, 19 and 27 nucleotides in *Hevea *whereas they have 21 and 24 nucleotides in other species. Indeed, in *Arabidopsis thaliana *[52], *Arachis hypogea *[45] and *Oryza sativa *[53], the fraction of 24 nucleotides has proved to be predominant, accounting for 60%, 45% and 36% of unique small RNAs, respectively. The predominant size has been found to be 21 nucleotides in *Pinus contorta *[53] and *Taxus chiniensis *[54]. In *Hevea*, the high percentage of 17- and 19-nucleotide small RNAs resulted from the increased amount of RNA degradation products and transposable elements in response to abiotic treatments according to the mapping results against the *Arabidopsis *genome (data not shown), as previously observed in *Brachypodium *[55]. Indeed, a change in small RNA size distribution has been reported in *Brachypodium *in response to cold treatment. Under normal growth conditions, the 21- and 24-nucleotide classes were the most abundant small RNA families, whereas it was the 19-nucleotide class after cold treatment [55]. Our dataset was generated from pooled small RNAs of various tissues subjected to several types of abiotic stress. The size distribution may have been differentially affected by each type of abiotic stress and may explain the difference in size distribution in *Hevea*.

Long conserved and novel miRNAs of 23-27 nucleotides consisted of short miRNA sequences with an extension. This lmiRNA structure suggested that they were generated from the same *MIR *genes. Our observation suggests dual functions for 14 canonical *MIR *genes producing 20-22 nt and 23-27 nt lmiRNA in *Hevea*, namely *HbMIR*169, *HbMIR*2911, *HbMIR*482, *HbMIR*472, *HbMIR*408 and *HbMIR*n3-10. Across the plant kingdom, lmiRNA were previously classed as heterochromatic small interfering RNAs (hc-siRNA) [28]. In *Arabidopsis*, the hc-siRNA derived from the miRNA-generating site is dependent on DCL3, RDR2 and PolIV associated with AGO4 and direct DNA methylation for some target sites [28]. As regards the higher number of reads for the 24 nucleotide compared with the 20-22 nucleotide miRNAs, the 14 miRNA families producing 20-22 nt and 23-27 nt miRNAs might mostly regulate their targets through direct DNA methylation in response to abiotic stress. This may confirm the results of a recent study showing the existence of site-specific abiotic stress-induced DNA methylation for five genes in *Hevea *[56].

### Ancient and recently differentiated *Hevea brasiliensis *miRNAs provide additional information for evolutionary studies

The 48 *Hevea brasiliensis *conserved miRNA families were more or less similar to those of other species. In *Hevea*, five miRNA families (HbmiR319, HbmiR156/157 and HbmiR165/166) have been found in more than forty plant species [57], and nine correspond to ancient miRNA already present in the common ancestor of Embryophytes (HbmiR156, HbmiR159/319, HbmiR160, HbmiR165/166, HbmiR171, HbmiR408 and HbmiR395), two in the common ancestor of Tracheophytes (HbmiR397 and HbmiR398) and nine in the common ancestor of Spermaphytes (HbmiR162, HbmiR164, HbmiR168, HbmiR169, HbmiR172, HbmiR393, HbmiR394, HbmiR399 and HbmiR827) [58]. Interestingly, the so-called monocotyledon-specific HbmiR444 family was identified in this study [58]. This suggests that HbmiR444 appeared before the speciation between monocotyledons and dicotyledons. Three woody species-specific miRNA families were also found in *Hevea brasiliensis*: HbmiR473 (*Citrus sinensis *and *Populus trichocarpa*), HbmiR476 (*Populus trichocarpa*) and HbmiR479 (*Populus trichocarpa, Vitis vinifera, Gossypium hirsutum *and *Citrus sinensis*) and were probably recruited independently by woody species during their evolution. The 10 novel miRNA families identified in this work are likely to be more recent, after *Hevea *diversification, as they have not been identified up to now in other euphorbiaceous plants. Furthermore, this information complements previous studies on euphorbiaceous species [49] in which only 25 conserved miRNAs were identified.

Our data could be used to study the evolution of *MIR *genes. We showed that 70% of the putatively new *MIR *genes were able to produce lmiRNA of 23-27 nucleotides, compared to 29% for conserved *MIR *genes. This corresponds to what has been observed in *Arabidopsis*, where it was previously suggested that young *MIR *genes consistently produced siRNAs, while ancient *MIR *genes produced predominantly canonical miRNAs, showing that the evolution of *MIR *genes is associated with changes in the use of DCL, resulting in specific miRNA size classes [59]. In addition, several so-called plant species-specific miRNAs were also found in *Hevea*, such as HbmiR1310 initially found only in *Pinus taeda *[53], HbmiR2910, HbmiR2914, HbmiR2915 and HbmiR2916 found in *Populus euphratica *[60] and HbmiR476 in *Populus trichocarpa *[61]. Our data suggest that those miRNAs should no longer be considered as species-specific miRNA.

Our study also revealed some conserved and divergent mechanisms at post-transcriptional level. Indeed, four families of miRNA were subjected to RNA editing events at the sites previously observed in *Arabidopsis*. However, for the thirteen families left, RNA editing was not observed under our experimental conditions. Under our experimental conditions, the -1 + UU modification was not observed in *Hevea *for the nine families selected from Ebhardt et al. [50]. Once the *Hevea *genome sequence is available, it will be possible to conduct an exhaustive analysis of the discrepancies between members of a multigenic family and RNA editing events.

### Predicted target/miRNA pairs conserved between plant species and putative new ones in *Hevea brasiliensis*

In comparison with other plants, several regulation mechanisms seem to be conserved in *Hevea brasiliensis*, such as the regulation of HD-ZIP III protein by HbmiR166 [62], Squamosa promoter binding protein by HbmiR156 [63], NAC domain protein by HbmiR164 [64], APETALA2-like protein by HbmiR172 [65-67], CCAAT-binding transcription factor by HbmiR169 and cysteine proteinase by HbmiR396 [44]. These ancient miRNAs regulate ancestral transcription factors that coordinate highly conserved functions, such as organ polarity and separation, cell division, or hormonal control [68,69]. We predicted potentially new miRNA/target pairs in *Hevea*. New target/miRNA pairs were found for transcription factors, such as, for example, the NAC domain protein by HbmiR165 and HbmiRn7, the GRAS domain protein by HbmiR170, HbmiR396 and HbmiRn5, WRKY by HbmiR396 and HbmiR444, and the APETALA2-like transcription factor by HbmiR156, HbmiR159, HbmiR393, HbmiR396 and HbmiR408. (Duan et al., submitted). In addition, we also detected new pairs involved in protein degradation (ubiquitin protein ligase by HbmiRn6 and HbmiRn7), in response to hormones (ABA-insensitive by HbmiR166, ABA responsive element binding protein by HbmiR2910, and HVA22-like protein by HbmiR398, CTR by HbmiRn5) and in the regulation of miRNA (cap binding protein by HbmiR167). Further analyses are needed to validate the inhibition of these targets at transcriptional or translational level and to unravel specific regulation in *Hevea brasiliensis *regarding the response to stress in latex cells, and latex production.

### Conserved and divergent regulation of redox homeostasis in *Hevea brasiliensis*

Several enzymes involved in ROS production or detoxification were found in our analysis. Two ROS-producing enzymes, Rboh, are likely to be targeted by HbmiR2914 and HbmiR476. They are key regulators in the ROS signalling network as they integrate different signal transduction pathways, such as calcium, protein phosphorylation and lipid signalling with ROS production [6]. Careful attention was also paid to targets classified in the GO term "antioxidant activity". For conserved miRNAs, two CuZnSOD chloroplastic and peroxisomal isoforms were predicted to be targeted by HbmiR398 and HbmiR159 respectively. Surprisingly, the CuZnSOD cytosolic isoform was not predicted by the LeARN pipeline and, even manually, no correct alignment was possible with HbmiR398. So far, validation by 5' RACE PCR has failed under our experimental conditions. By contrast, previous studies in *Arabidopsis *showed that HbmiR398 targeted both chloroplastic and cytosolic isoforms [34].

Some miRNA families have been reported to be H_2_O_2_-responsive in rice, such as OsmiR169, OsmiR397, OsmiR528, OsmiR827, OsmiR319 and OsmiR408 [70]. In rice, OsmiR397 targets laccase genes involved in lignin biosynthesis and those targets were also found for HbmiR397 in *Hevea*.

## Conclusion

A library of small RNAs was deeply sequenced to identify conserved and novel miRNAs in *Hevea brasiliensis*. Combined with the *Hevea *transcript database, this led to the identification of 48 miRNA families and 10 novel miRNAs. Our study revealed new insights for evolutionary studies on woody plants and the potentially specific regulation of redox homeostasis in *Hevea*. All the putative targets of interest for *Hevea *stress biology and latex production will be experimentally validated and the target genes and their corresponding miRNAs will undergo co-expression analysis.

## Methods

### Production of in vitro plantlets

Embryogenic friable callus from line CI05538 of clone PB 260 was maintained on MM medium in the culture room at 27°C, with 60% humidity in the dark [71]. In vitro plants were regenerated according to the procedure described in [72]. Briefly, somatic embryogenesis was initiated by sub-culturing 1 gram of callus in 250 mL flasks containing 50 mL of a semi-solid Induction Medium. Pro-embryos were then developed in a temporary immersion system (RITA^®^, CIRAD, France) with 1 min of immersion per day in the liquid development media DEV1 and DEV2. For plant regeneration, only well-shaped mature embryos were collected after 8 weeks of culturing [73]. They were transferred to glass tubes on a semi-solid DEV3 medium for one month under a light intensity of 60 μmol m^-2 ^s^-1 ^and a 12 h light/dark photoperiod for embryo conversion into plants. Plantlets were then acclimatized in the greenhouse at 28°C with 60% relative humidity.

### Plant material cultivation and treatments

Budded plants and two-year-old in vitro plants of clone PB260 were grown in the greenhouse at 27°C, with 50% humidity under natural light conditions. Budded plants and two-year-old in vitro plants of clone PB260 were exposed to cold at 4°C for 8 h, light (PAR = 1000-1500 μmol/m^2^/s for 4 h), drought (4 weeks for young budded plants and 10 days for two-year-old in vitro plants without watering), flooding (covered with water for 4 days), salinity (300 mM NaCl for 4 days) and wounding for 4 h (Table 2). Hormonal treatments were applied in hermetically sealed boxes, either with 5 ppm of ethylene for 4 h or 0.3 μM of methyl jasmonate for 4 h as described in [74,75].

### Isolation of small RNAs

For all treatments, leaves, bark and roots were sampled in liquid nitrogen and then frozen at -80°C pending small RNA isolation. Small RNAs were purified with the mirPremier microRNA Isolation Kit (Sigma-Aldrich, St. Louis, MO, USA), following the manufacturer's instructions for plant tissues. Briefly, 100 mg of the plant tissue powder was mixed with a lysis mix that released small RNA and, at the same time, inactivated ribonucleases and interfering secondary metabolites that might exist in plant tissues. Large RNA and genomic DNA remained insoluble and were removed from the lysate along with other cellular debris by brief centrifugation. Small RNA was then captured on a silica binding column in the presence of alcohol. Residual impurities were removed by wash solutions, and purified small RNA was eluted in RNase-free water.

### Deep sequencing of small RNAs

Small RNA samples from callus and from control and treated plants were pooled. A *Hevea *small RNA library was constructed by the SKULTECH Company (France). Briefly, small RNAs were separated on a denaturing 15% polyacrylamide gel. The gel slices containing RNA with a size of about 15-50 nucleotides were excised and the RNA was eluted. Small RNAs were ligated to 5' and 3' adapters using T4 RNA ligase. Products from the second ligation were gel purified then reverse transcribed and amplified using PCR to produce cDNA. The PCR was performed with two primers that annealed to the ends of the adapters. PCR products were separated on 15% polyacrylamide gel with ethidium bromide staining. The gel slices containing DNA with a size of about 92 nucleotides were excised and the DNA was eluted. The resulting cDNA was sequenced using Solexa/Illumina technology (Illumina Inc., San Diego, CA, USA). Raw reads were trimmed using Trim_Adaptor application (non Open source application) with the minimum clip length fixed at 6 nucleotides.

### Bioinformatic analyses

This work was supported by the high performance cluster of the SouthGreen Bioinformatics platform (southgreen.cirad.fr - UMR AGAP - CIRAD) comprising 26 compute nodes (208 Nehalem cores) and a 16 to storage capacity connected through a low latency Infiniband network. Sun Grid Engine was used as the workload manager to schedule jobs on the different compute nodes.

### Identification of the conserved *hevea *miRNAs

Adapters and low-quality sequences were removed from the raw reads obtained from Illumina sequencing using cross-match software http://www.phrap.org/phredphrpconsed.html; the non-redundant dataset was produced using WU nrdb software. The unique small RNA sequences were then compared with BlastN [76] against the *Arabidopsis *genome (Phytozome V6) and the PMRD database [38]http://bioinformatics.cau.edu.cn/PMRD in order to classify the small RNAs and identify mature miRNAs in *Hevea *already known in several species.

### Identification of *Hevea *miRNA precursors

Conserved miRNAs and putative novel miRNA precursors were identified in the PB260 transcriptome http://bassigny/cgi-bin/esttik_dev/quick_search.cgi using the LeARN pipeline http://symbiose.toulouse.inra.fr/LeARN/release 1;5.0[50], a platform for detecting, clustering and annotating non-coding RNAs. Novel miRNAs were separated into 4 classes according to their position on the stem-loop structure. Class 1 precursors only produced small RNAs corresponding to the predicted miR and miR* (i.e., small RNAs in a 25-bp region around the miR:miR* predicted region to be considered with two nucleotide overhangs linked to DCL action); class 2 comprised hairpins containing both predicted miR and miR* and additional small RNAs, of lower abundance, outside this region; class 3 involved precursors with reads on the defining small RNA region only (3a, at least two reads; 3b, only one read); class 4 consisted of precursors containing the defining small RNA plus reads of lower abundance outside the predicted miR:miR* duplex. Finally, class 5 contained hairpins that generated small RNAs of greater abundance than the defining RNA outside the predicted miR:miR* region [77]. Each miRNA precursor was checked manually following the rules described in [45]. Specifically, we considered dominant, mature sequences residing in the stem region of the stem-loop structure and ranging between 20-22 nt with a maximum free-folding energy of -25 kcal mol^-1^. A maximum of six unpaired nucleotides between the miRNA and miRNA* was allowed. The distance between the miRNA and miRNA* was fixed between 5 and 240 nucleotides.

### Identification of putative *Hevea *miRNA targets

Computational identification of miRNA targets was performed with the Miranda toolbox included in the pipeline with the default parameters (gap_value = 2, mm_value = 1, gu_value = 0.5, score_threshold = 3, min_length_alignment = 20 and no_mismatch_positions = 10;11) for conserved miRNA and secondly checked with psRNAtarget http://plantgrn.noble.org/psRNATarget/(length_alignment = 18), [78]. For the putative novel miRNAs, psRNAtarget server (length_alignment = 18) and Miranda (gap_value = 2, mm_value = 1, gu_value = 0.5, score_threshold = 3, min_length_alignment = 18 and no_mismatch_positions = 10; 11) were used to scan the *Hevea *EST databases (Duan et al., to be submitted, http://bassigny/cgi-bin/esttik_dev/quick_search.cgi). A first assembly set was generated from reads of leaves, bark, latex, embryogenic tissues, and roots separately to create tissue-specific transcript databases with sequence names CLxxcontigxx. Then, reads from all tissues were used to generate one transcript sequence database for the *Hevea *clone PB260 with the sequence name hevea_454_xxx.

Gene Ontology analyses were performed with the Blast2GO program [79,80]. In detail, sequence analyses, such as alignments and BLAST, were performed with Geneious software [81].

### Total RNA extraction

Total RNAs from leaves, bark and roots subjected to abiotic stress treatments were extracted using the caesium chloride cushion method adapted from Sambrook [82] by Duan et al. [75]. One gram of fresh matter was ground and transferred to a tube containing 30 ml of extraction buffer consisting of 4 M guanidium isothiocyanate, 1% sarcosine, 1% polyvinylpyrrolidone and 1% ß-mercapto-ethanol. After homogenization, the tubes were kept on ice and then centrifuged at 10,000 g at 4°C for 30 minutes. The supernatant was transferred to a new tube containing 8 ml of 5.7 M CsCl. Ultracentrifugation in a swinging bucket was carried out at 89,705 *g *at 20°C for 20 hours. The supernatant and caesium cushion were discarded, whilst the RNA pellet was washed with 70% ethanol. After 30 minutes of air drying, the pellet was dissolved in 200 μl of sterile water. RNAs were conserved at -80°C.

### Experimental validation of miRNA targets

The GeneRacer kit (Invitrogen, Carlsbad, CA, USA) was used to ligate a 5'RNA adapter to pooled RNAs prior to reverse transcription. The resulting cDNAs were used as a template for PCR amplification using one Gene-Specific Primer (GSP) designed downstream of the predicted miRNA:target binding site and one GeneRacer 5' forward primer. The primers used were HbCuZnSODchl R: GGGTAACCAGCAAATGCAAGCAGC, HbARF R: CTTGTGTTGAGTTGCAGCGCG and HbSquamosa R: TGGAGCCTAATTGGCTTCCTTCTGC. The first PCR product was then used for nest amplification performed with a nested GSP primer and a nested GeneRacer 5' forward primer. The nested primers used were HbCuZnSODchl nested R: GCAGGGAACAATGGCTGCC, HbARF nested R: ATGTTCAGCTCAGTGGACACGG, and HbSquamosa nested R: TTGTTTGGCACCACGCTTGTGGAAG. The PCR products were gel purified, cloned into PCR2.1 TOPO TA vector (Invitrogen, Carlsbad, CA, USA) and sequenced.

### Complementary DNA (cDNA) synthesis and stem-loop RT-PCR

We checked for the absence of contaminating genomic DNA prior to cDNA synthesis. If genomic DNA was detected, a DNAse treatment was performed using TurboDNAse (Ambion, Texas, USA) following the manufacturer's instructions. Four micrograms of DNA-free RNAs was used for cDNA in a 40 μL reaction mixture using a RevertAid™ M-MuLV reverse transcriptase following the manufacturer's instructions (MBI, Fermentas, Canada). The primers used for the stem-loop amplification are presented in Table 9. RT-PCR cycling conditions comprised one denaturation cycle at 95°C for 2 min, followed by 35 amplification cycles (95°C for 20 s, 58°C for 30 s, and 72°C for 30 s). All primers were also validated by real-time PCR. The real-time PCR reaction mixtures consisted of 2 μL of RT product cDNA, 1 μL of 5 μM of each primer, and 3 μL 2 × SYBR green PCR master mix (LightCycler^® ^480 SYBR Green I Master, Roche Applied Sciences) in a 6 μL volume. PCR cycling conditions comprised one denaturation cycle at 95°C for 10 min, followed by 45 amplification cycles (95°C for 5 s and 60°C for 20 s). Melting curves were analysed to check the specificity of the PCR amplification (Table 9). The standard curve was generated using a two-fold dilution series of 10 points in triplicate from a mixed cDNA sample. This standard curve allowed the calculation of primer efficiencies (Table 9).

**Table 9:** Primer list for the stem-loop RT-PCR and the real-time PCR and their efficiencies

Precursor name	Forward primer	Reverse primer	Unique melting pick	PCR efficiency
Hbpre-miR156	TGGTGATGTTGTTGACAGAAGATAGAGAGC	GCACAAAGGAGTGAGATGCAGAGTCC	Yes	1,790

Hbpre-mir159a	GGTTAAGAAGTGGAGCTCCTTGAAGTC	GCTCCCTTCAATCCAAACAAGGATC	Yes	1,958

Hbpre-mir159b	GTGGAGCTCCTTGAAGTCCAATAGAGG	AGAGCTCCCTTCAATCCAAACAAGG	Yes	1,881

Hbpre-mir166b	GGGGAATGTTGTCTGGTTCGATG	TCAAATCAAACCCTGTTGGGGG	Yes	1,738

Hbpre-mir396	TGACCCTCTTCGTATTCTTCCACAGC	CCCACAGCTTTATTGAACCGCAAC	Yes	1,782

Hbpre-mir408b	GACATACAAAGACTGGGAACAGGCAG	GCCACAAGCCAGGGAAGAGGC	Yes	1,792

Hbpre-mir476	GCCTTGTATGTTTCATTTAGTAATCCTTCT	GATAATCCTTCTATGCAAAGTCTTTTATGC	Yes	1,732

Hbpre-mir2910	CAGGTCCAGACATAGTAAGGATTGACAG	CGTTAACGGATTAACCAGACAAATCGC	Yes	1,728

Hbpre-miRn1	ACCAGGAACTGGTATCAACCCAGC	TGCTACCAATGAATCGGACCCACC	Yes	1,837

Hbpre-miRn2	CAGTAAATAGCAGTATCGTGGATAGGG	GTCCAATCATTGATCCTGAAAATTTCTAC	Yes	1,828

Hbpre-miRn3	TGGATTGGAGCCCAATACTGTGAC	CTGCTCCATTGATTTTACCATCTATGC	Yes	1,873

Hbpre-miRn4	TGCGTGGGTAGATTGAGCTGC	GCTCCTGAGATATGCAAGCCACAAG	Yes	1,863

Hbpre-miRn6	ACCTAGGATGTAGAAGAGCATAAC	ACTACATGAGTGGATATATAGGAATCC	Yes	1,787

Hbpre-miRn7	ATCTGCTACTCGGATAACCGTAG	GCAGCAAATCTTCCATAGCATCC	Yes	1,925

Hbpre-miRn8	TTTCTTGATTCTAGTGGGTGGTG	GGAATTAACCAGACAAATCGCTC	Yes	1,905

Hbpre-miRn9	AGTCGGGGGCATTCGTATTTC	GCAAATACTTTCGCAGTTGTTCG	Yes	1,956

Hbpre-miRn10	TGGATTTATGAAAGACGAACAACTGCG	TTCGAGCCCCCCAATTTCG	Yes	1,712

## Abbreviations

miRNAs: microRNAs; TPD: Tapping Panel Dryness; ROS: Reactive Oxygen Species; PMRD: Plant microRNA database; DCL1: RNAse III-like DICER-like I; HASTY: plant ortholog to exportin 5; RISC: RNA-induced silencing complex; AGO1: Argonaute 1; EST: Expressed Sequence Tag

## Authors' contributions

GV, AX, AW and PB carried out the bioinformatics studies. AX carried out the EST assembly and managed work with the LeARN pipeline. PB installed the cluster and made all the IT changes to adapt the LeARN program to the cluster. DC provided *AP2/ERF *sequences from *Hevea*. GV, MP and LJ drafted the manuscript. MP and LJ devised the research project and took part in its design and coordination. All the authors read and approved the final manuscript.
